# Neonatal mortality in Ethiopia: trends and determinants

**DOI:** 10.1186/1471-2458-13-483

**Published:** 2013-05-17

**Authors:** Yared Mekonnen, Biruk Tensou, Daniel S Telake, Tedbabe Degefie, Abeba Bekele

**Affiliations:** 1 Mela Research PLC, P.O. Box 34422, Holy City Center Building, 4th Floor, Addis Ababa, Ethiopia; 2 Save the Children, USA, P.O. Box 387, Addis Ababa, Ethiopia; 3 UNICEF Ethiopia, P.O. Box 1169, Addis Ababa, Ethiopia

**Keywords:** Ethiopia, Neonatal mortality, Trends, Determinants

## Abstract

**Background:**

The Ethiopian neonatal mortality rate constitutes 42% of under-5 deaths. We aimed to examine the trends and determinants of Ethiopian neonatal mortality.

**Methods:**

We analyzed the birth history information of live births from the 2000, 2005 and 2011 Ethiopia Demographic and Health Surveys (DHS). We used simple linear regression analyses to examine trends in neonatal mortality rates and a multivariate Cox proportional hazards regression model using a hierarchical approach to examine the associated factors.

**Results:**

The neonatal mortality rate declined by 1.9% per annum from 1995 to 2010, logarithmically. The early neonatal mortality rate declined by 0.9% per annum and was where 74% of the neonatal deaths occurred. Using multivariate analyses, increased neonatal mortality risk was associated with male sex (hazard ratio (HR) = 1.38; 95% confidence interval (CI), 1.23 − 1.55); neonates born to mothers aged < 18 years (HR = 1.41; 95% CI, 1.15 − 1.72); and those born within 2 years of the preceding birth (HR = 2.19; 95% CI, 1.89 − 2.51). Winter birth increased the risk of dying compared with spring births (HR = 1.28; 95% CI, 1.08 − 1.51). Giving two Tetanus Toxoid Injections (TTI) to the mothers before childbirth decreased neonatal mortality risk (HR = 0.44; 95% CI, 0.36 − 0.54). Neonates born to women with secondary or higher schooling vs. no education had a lower risk of dying (HR = 0.68; 95% CI, 0.49 − 0.95). Compared with neonates in Addis Ababa, neonates in Amhara (HR: 1.88; 95% CI: 1.26 − 2.83), Benishangul Gumuz (HR: 1.75; 95% CI: 1.15 − 2.67) and Tigray (HR: 1.54; 95% CI: 1.01 − 2.34) regions carried a significantly higher risk of death.

**Conclusions:**

Neonatal mortality must decline more rapidly to achieve the Millennium Development Goal (MDG) 4 target for under-5 mortality in Ethiopia. Strategies to address neonatal survival require a multifaceted approach that encompasses health-related and other measures. Addressing short birth interval and preventing early pregnancy must be considered as interventions. Programs must improve the coverage of TTI and prevention of hypothermia for winter births should be given greater emphasis. Strategies to improve neonatal survival must address inequalities in neonatal mortality by women's education and region.

## Background

Neonatal mortality, accounting for an estimated 4 million deaths worldwide each year, constitutes 40% of under-5 mortality and approximately 57% of infant mortality [1]. Most neonatal deaths (99%) arise in low-income and middle-income countries, and approximately half occur at home [2]. In the past two to three decades, neonatal mortality rates have shown a slow decline in developing countries whereas infant and under-5 mortality rates have declined significantly [1,3-6].

With a population of nearly 83 million in 2010 [7], Ethiopia is the second most populous country in Africa after Nigeria. The population grows at a rate of 2.6% per annum and the majority of people (84%) reside in rural areas, with agriculture being the major source of livelihood [8]. High mortality, high fertility and low life expectancy characterize the demography, as in most sub-Saharan African countries. In the past decade, however, the country witnessed an unprecedented decline in under-5 mortality from 166 per 1000 in 2000 to 88 per 1000 live births in 2011 [9], an average decline of 47%.

Approximately 42% of the under-5 mortality in Ethiopia is attributable to neonatal deaths [9]. According to the 2011 Ethiopia Demographic and Health Surveys (DHS), the country is experiencing a high neonatal mortality rate at 37 per 1000 live births, comparable to the average rate of 35.9 per 1000 live births for the African region overall [10]. The causes of neonatal mortality are not well documented in Ethiopia, but previous studies report causes such as sepsis, asphyxia, birth injury, tetanus, preterm birth, congenital malformations and unknown causes [7].

Over the last decade, neonatal deaths have gained importance on the world policy agenda because the Millennium Development Goal (MDG) for child survival cannot be met without substantial reductions in neonatal mortality. It is estimated that reduction of under-5 child mortality by two-thirds by 2015, as called for by the MDG, requires a reduction in neonatal mortality of at least 50% [4]. There are highly feasible and cost-effective interventions that could avert up to 72% of neonatal deaths [11], but this can only be achieved if countries adopt locally relevant and focused interventions that are guided by evidence.

Many studies have shown that neonatal mortality is influenced by multiple factors [2,12-14]. Maternal health before, during, and after pregnancy, conditions at the time of labor and delivery and postnatal care of babies play a significant role in reducing neonatal mortality [11]. Socioeconomics, demographics, the health care system, cultural practices and technology are also important indirect determinants of neonatal mortality [2,13,14].

There is a general dearth of studies on neonatal mortality in Ethiopia, which significantly limits our understanding of the breadth and depth of the problem for evidence-based programming. We aimed to contribute to the understanding of the trends and determinants of neonatal mortality in Ethiopia using data from the 2000, 2005 and 2011 Demographic and Health Surveys.

## Methods

### The Ethiopia Demographic and Health Surveys (EDHS) data

The first DHS was conducted in Ethiopia in 2000; followed by 2005 and 2011. The surveys were based on nationally representative probability samples that covered the entire country. Women aged 15–49 years and men aged 15–59 years were interviewed using standard questionnaires. The three surveys collected data from mothers or caretakers of live-born infants in the 5 years preceding the date of the interview. Socioeconomic and demographic information was also collected from women and households. The data from the three surveys were pooled for analysis. Details about the Ethiopia DHS procedure and methodology can be obtained from the national reports [9,15,16]. Our analysis was based on 32 428 live-born infants (32 042 singletons and 386 multiple births) in the 5 years preceding the date of the interview.

### Data availability

The DHS data used for this study are openly available and can be downloaded from: http://www.measuredhs.com/data/available-datasets.cfm?inputSearch=ETHIOPIA. We also obtained the full data sets for the three surveys on a CD-ROM from the Central Statistical Agency of Ethiopia.

### Study variables

The inclusion of variables was partly guided by the Mosley and Chen conceptual framework previously used in similar studies [17]. The original Mosley and Chen conceptual framework was not fully implemented in this study because of inadequate data. We employed a reduced form of the framework that has been used elsewhere [18,19]. The factors influencing neonatal mortality were broadly categorized as contextual, socioeconomic, proximate factors and maternal health service use.

Early neonatal mortality rate (ENMR) was defined as the probability of dying before 7 completed days of life; late neonatal mortality rate (LNMR) as the probability of dying between 7 completed days and before 28 completed days; and overall neonatal mortality rate (NMR) as the probability of dying before 28 completed days.

Estimates of neonatal mortality were based on information collected in the DHS birth history section of the questionnaire administered to individual women and were based on the reported births occurring in the previous 5 years. Based on the 2000 survey, the earliest date of birth of children included in this analysis is 1995. For each live birth to a woman, questions on the sex of the child, year and month of birth, whether the child is alive or dead, if dead the age of the child at the time of death were asked and recorded. If a child died before the age of 1 month, this was recorded in days (i.e. age in days at the time of death). The NMR, ENMR, and LNMR were estimated directly from the information in the birth history from a child’s birth date, survivorship status, and age at death for children who died.

The contextual factors included in this analysis were urban–rural residence and region. The socioeconomic factors included maternal education, household wealth and marital status. We used three categories for women's educational status: no education, elementary education (1–6 years of schooling), and secondary or higher education (7 plus years of schooling). The DHS raw data were provided with wealth index variables that were constructed to rank households using principal component analysis. The wealth quintiles were trichotomized into low, medium and high wealth scores. Marital status was dichotomized as currently married and not currently married.

The proximate factors included inherent child characteristics, fertility patterns, and season of birth. The specific variables were child's sex; size of child at delivery; preceding birth interval; birth order; age of the mother at childbirth; and season of birth. The size of a child at birth was based on the mother's or caretaker's report in five categories, which were then collapsed into two categories: smaller than average vs. average/larger than average for the purpose of our analysis. The birth interval variable was trichotomized as follows: preceding interval less than 2 years; preceding interval 2 or more years and only one birth (not applicable). Birth order was also trichotomized as: first-/second-born; third-born; and fourth or higher order. The categorizations of preceding birth interval and birth order were based on previous literature in Ethiopia [9] and elsewhere [18,19]. Women's age pattern of fertility was assessed using the mother's age at child birth, which was categorized as: less than 18 years, 18–34 years, and 35–49 years. The date of birth of the live-born infants was collapsed into four Ethiopian seasons: spring (September to November), summer (December to February), autumn (March to May) and winter (June to August).

A small number of variables were examined in relation to maternal health service use. These were the mother's receipt of TTI before birth (categorized as two TTIs vs. none or one TTI); place of delivery/assistant during delivery; and mode of delivery (vaginal vs. caesarian section). Five categories were created for the delivery variables: (1) home delivery assisted by families/friends; (2) home delivery assisted by traditional birth attendants (TBA)/health workers; (3) government hospital; (4) health center; and (5) private/non-governmental organization (NGO) facilities. Antenatal care use in the DHS was collected only for the most recent births and thus not included in this analysis.

### Method of analysis

Trends in the overall neonatal mortality as well as the early and late neonatal mortality rates during the period 1995–2010 were examined using simple linear regression models after transforming the neonatal mortality rates to a logarithmic scale. To examine the determinants of neonatal death, univariate and multivariate analyses were performed using the Cox proportional hazards regression model. The univariate models provided the unadjusted hazard ratio (HR) and the multivariate adjusted HR.

The multivariate analyses were constructed using a hierarchical modeling approach [20], as in previous studies [18,19]. Potential independent variables were entered into the model progressively and step by step from distal factors to the proximate factors. We used a backward elimination procedure, requiring a variable to reach p < 0.05 to remain in the model. The initial model included only the contextual factors (residence, region and year of birth). This was followed by a backward elimination of variables. Socioeconomic factors (mother's education, household wealth and marital status) were added to the variables retained from the initial model. This was also followed by a backward elimination. The third step added proximate factors i.e., child's sex; birth interval; birth order; size of child at birth; mother's age at birth; and season of birth into the model based on contextual and socioeconomic factors. As before, this was followed by a backward elimination. Finally, maternal health service-related factors (the receipt of two TTIs, place of delivery, and mode of delivery) were added. We included the unadjusted HRs with 95% CIs for all 15 variables examined in this study, while adjusted HRs are shown only for those factors that were retained in the final model. A 95% CI that did not contain the null value, 1, indicated statistical significance. We also examined the interaction effects of certain variables and found a significant interaction between place of delivery and urban–rural residence. As a result, we presented a separate model for the urban and rural samples to examine the effect of place of delivery on the risk of neonatal death.

We used Stata version 10 (Stata Corporation, College Station, TX, USA) for data management and analyses. The Survey command in STATA was used to declare the strata and primary sampling unit. All of the proportions, rates and hazard ratios were weighted for the sampling probabilities.

### Assessment and quality of data

The trend analyses are based on a direct estimation of neonatal mortality rate using birth history data. The reliability of the neonatal mortality rate estimates depends on the accuracy and completeness of reporting and recording of births and deaths. We examined the sampling and non-sampling errors associated with the data used for the direct estimation of the neonatal mortality rate.

**Figure 1:**
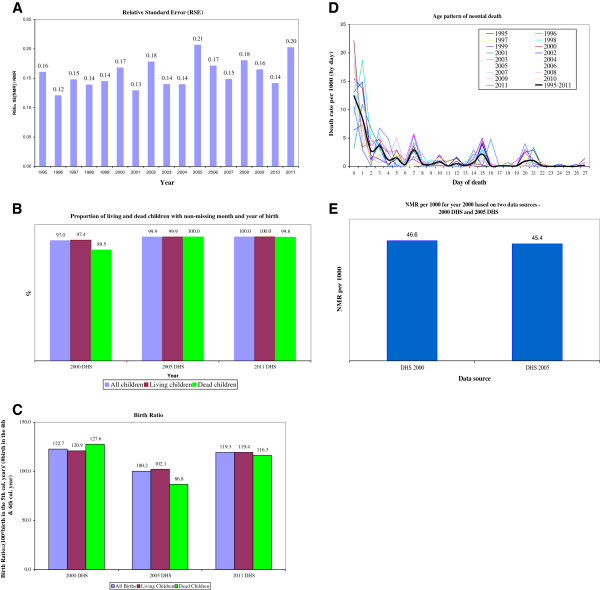
**Indicators of completeness and quality of the Ethiopian neonates birth history data: 2000, 2005 and 2011 DHS.** (**A**) Relative standard error. (**B**) proportion of living and dead children with non-missing month and year of birth. (**C**) birth ratio. (**D**) Age pattern of neonatal death. (**E**) NMR for year 2000 based on two data sources- 2000 and 2005 DHS.

To determine the sampling error associated with the neonatal mortality estimates, we computed relative standard error (RSE) [21] of the rate by year (Figure 1A). The RSE, also known as the coefficient of variation, is computed by dividing the standard error of the estimate by the mean of the estimate itself multiplied by 100. One of the advantages of using RSE as a measure of reliability is that, because it is scale-less, it permits comparisons with other estimates that are measured in entirely different units. It is suggested that estimates having RSEs exceeding 25% of the threshold are less reliable. Some researchers suggest that estimates with RSEs exceeding 35% should not be used to draw statistical inferences and should not be released to the public [22]. As shown in Figure 1A, the RSE estimates for the neonatal mortality rates by year range between 12% and 21%, suggesting that the estimates are within acceptable standard error.

The month and year of birth of live-born infants included in this analysis can be considered complete for both living and dead children (Figure 1B), with the exception of the year 2000 data where 10.5% of dead infants had a missing month or year of birth. For the other years, the month and year of birth were complete for over 97% of the living and dead children.

Omission and misclassifications of births in the 5th year preceding the survey are one of the problems of the DHS surveys [21]. We calculated the birth ratio of the number of births in the 5th calendar year to the sum of the births in the 4th and 6th calendar years to check for the presence or omission of births in the furthest reference period, as suggested by Curtis [21]. A birth ratio for the 5th calendar year equal to or greater than 100 suggests the lack of omission of births. As shown in Figure 1C, omission of births is evident for the 2005 DHS where the birth ratio for the dead children in the 5th preceding year was approximately 87, which is 13 points lower than the expected 100 points in the absence of omission. With the exception of dead children in the 2005 DHS, there was no evidence of birth omission for living and dead children in the other DHS data.

We examined the distribution of neonatal deaths by age (in days) for each calendar year (Figure 1D). The purpose of this analysis was to check for internal consistency of the age distribution of deaths among neonates from the different years. The distribution of neonatal deaths by age (in days) has a typical pattern, peaking in the first 2 days after birth and leveling off afterwards. Despite erratic patterns for some years, this pattern holds for most of the years, suggesting internal consistency of the data.

There was some overlap of reference periods for the 2000 and 2005 DHS. Both surveys collected birth history data for part of the 2000 calendar year. This created an opportunity to compare neonatal mortality rates for 2000. As shown in Figure 1E, the neonatal mortality based on the 2000 DHS compares well with the 2005 DHS data at 46.6 and 45.4 per 1000, respectively, with overlapping confidence intervals. This supports the external consistency of the survey data.

Overall, our assessment of the sampling and non-sampling errors of the survey data suggest that the birth history data are within the acceptable range and are fairly robust for the direct estimation of neonatal mortality as well as examination of trends over time.

## Results

### Characteristics of the study population

The three surveys collected data from mothers or caretakers of 32 428 live-born infants (32 042 singletons and 386 multiple births) in the 5 years preceding the date of the interview. On average, 1908 live births per year were reported in the three surveys between 1995 and 2011.

Table 1 presents selected child, maternal, household and contextual characteristics of the live-born infants. The live births included in this study were almost equally divided by sex. Slightly greater than one-third of the births were either first or second order births, while fourth or higher order births constituted approximately half (51.2%) of the total births, indicating high fertility. The distribution of the live births according to the preceding birth interval showed that 19.9% were first-born and 17.5% were born within less than 2 years of the preceding birth. Over 78% of the live births were born to mothers whose age was 18–34 years at childbirth. The vast majority of the births (88.8%) occurred at home. Over 76% of the live births were to mothers who did not have any formal education. Most women (> 89%) lived in the rural areas, reflecting the country's demographics.

**Table 1:** Characteristics of live births in Ethiopia from 1995–2011

	**N**	**%**
	**[Un-weighted]**	**[weighted]**
**Year of birth**		
1995-2000	10827	33.4
2001-2005	8927	27.5
2006-2011	11674	36.0
Missing	1000	3.1
**Birth type**		
Singleton	32042	99.0
Twin+	386	1.0
**Child’s sex**		
Male	16571	51.6
Female	15857	48.4
**Size at birth**		
Average or larger than average	22280	68.7
Smaller than average	9993	30.8
Missing	155	0.5
**Birthorder**		
1-2	11936	34.7
3	4613	14.1
4+	15879	51.2
**Birth interval**		
Not applicable (only one birth)	6443	19.9
<2 years	5668	17.5
2+ years	20276	62.5
Missing	41	0.1
**Age of mother at birth**		
<18 years	1947	5.5
18-34 years	25576	78.4
35+ years	4905	16.2
**Season of birth**		
Spring [Sept.-Nov.]	9208	27.1
Summer [Dec.-Feb.]	8592	25.2
Autumn [Mar.-May]	8031	25.4
Winter [June-Aug.]	6597	22.3
**Mother received Tetanus Toxoid Injection (TTI)**		
None or 1 TTI	26293	83.0
2+ TTI	6135	17.0
**Delivery place/assistance**		
Home delivery/not assisted by TBA/HW	20149	62.1
Home delivery - assisted by TBA/HW	8662	26.7
Government hospital	2044	6.3
Health center	1026	3.2
Other facilities (private/NGO)	547	1.7
**Mode of delivery**		
Non-caesarian section	31825	98.1
Caesarian section	603	1.9
**Mother's education**		
No education	24519	76.5
Elementary	5877	19.3
Secondary+	2032	4.2
**Household wealth score**		
Low	14203	43.8
Medium	6032	18.6
High	12193	37.6
**Marital status**		
Not currently married	2439	7.1
Currently married/in union	29989	92.8
**Residence**		
Urban	5067	10.6
Rural	27361	89.4
**Region**		
Tigray	3302	6.4
Affar	2348	1.1
Amhara	4377	23.6
Oromiya	5892	41.4
Somali	2368	2.5
Benishangul Gumuz	2529	1.1
SNNP	4939	21.7
Gambela	1974	0.3
Harari	1733	0.2
Addis Ababa	1296	1.5
Dire Dawa	1670	0.3
Total	32428	100.0

*SNNP* Southern Nations, Nationals and Peoples.

### Trends in neonatal mortality rate

**Figure 2:**
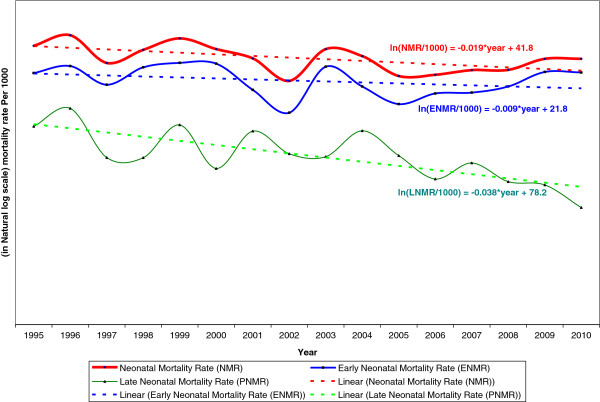
Trends in Neonatal, Early and Late Neonatal Mortality rate (in log scale): 1995–2010, Ethiopia.

**Figure 3:**
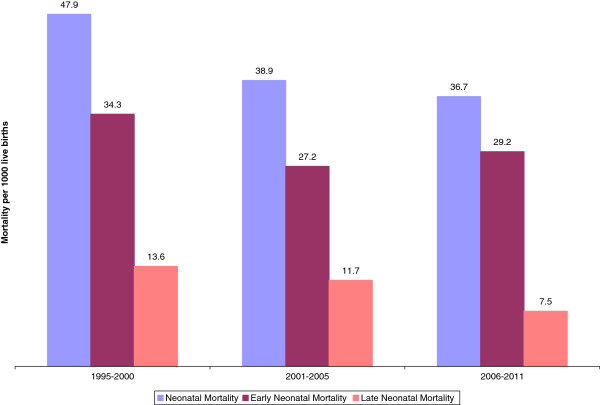
Ethiopian neonatal, early neonatal and late neonatal mortality rates by year.

Figure 2 presents the trend data for the NMR, ENMR and LNMR for the period 1995–2010. The mortality rate for each year was converted to a logarithmic scale and a simple linear regression model using year and neonatal mortality rate (in a logarithmic scale) was fitted separately for the three parameters. The data for year 2011 were not included in the analyses because of small sample size. Logarithmically, the neonatal mortality rate showed a slow decline of 1.9% per annum during the period. The decline for the log of early neonatal mortality rate was even slower at 0.9% per annum during the same period. The rate of decline was much faster for the log of the late neonatal mortality rate at 3.8% per annum. The trend for the overall neonatal mortality rate tilted heavily toward the trend of the early neonatal mortality rate because over 70% of the neonatal deaths occur in the first week of life (i.e., in the early neonatal period). Figure 3 shows that the relative contribution of the early neonatal mortality to the overall neonatal mortality increased from approximately 70% in 1995–2000 to 79.5% in 2006–2010. Therefore, the modest declining trend noted for the overall neonatal mortality in the country is mainly attributable to the decline in the post neonatal period.

### Determinants of neonatal deaths

The analyses of the determinants of neonatal deaths was restricted to singleton live-born infants (n = 32 042). We presented both univariate and multivariate results in Table 2. Unadjusted Hazard Ratios (HRs) are presented for all of the variables studied while adjusted HRs are presented only for those factors that were significantly associated with neonatal death in the multivariate analyses.

**Table 2:** Cox proportional hazard models for estimating neonatal mortality in Ethiopia by selected characteristics

	**Unadjusted**			**Adjusted**		
	HR*	**95% CI**		HR*	**95% CI**	
		**Lower**	**Upper**		**Lower**	**Upper**
**Child's year of birth**						
An increase of 1 calendar year	0.98	0.97	0.99	0.99	0.98	1.00
Contextual factors:						
**Residence**						
Urban (ref)	1.00					
Rural	**1.22**	1.03	1.46			
**Region**						
Tigray	**1.53**	1.04	2.26	**1.54**	1.01	2.34
Affar	0.98	0.64	1.51	0.86	0.54	1.37
Amhara	**1.94**	1.34	2.82	**1.88**	1.26	2.83
Oromiya	**1.63**	1.13	2.35	1.50	0.90	2.24
Somali	1.25	0.83	1.90	1.05	0.67	1.65
Benishangul Gumz	**1.90**	1.29	2.81	**1.75**	1.15	2.67
SNNP	1.32	0.90	1.93	1.30	0.86	1.96
Gambela	**1.56**	1.03	2.36	1.52	0.98	2.35
Harari	1.33	0.86	2.05	1.18	0.75	1.86
Dire Dawa	0.92	0.58	1.48	0.86	0.53	1.41
Addis Ababa (ref)	1.00			1.00		
Socioeconomic factors:						
**Mother's education**						
No education (ref)	1.00			1.00		
Elementary	**1.05**	0.91	1.22	1.07	0.92	1.25
Secondary+	**0.65**	0.49	0.87	**0.68**	0.49	0.95
**Marital status**						
Not currently married (ref)	1.00					
Currently married	**0.81**	0.66	0.99			
**Household wealth index score**						
Low (ref)	1.00					
Medium	1.04	0.91	1.20			
High	**0.81**	0.69	0.94			
Maternal/neonatal factors:						
**Child’s sex**						
Female (ref)	1.04					
Male	**1.42**	1.26	1.59	**1.38**	1.23	1.55
**Season of birth**						
Spring (ref)	1.00			1.00		
Summer	1.10	0.94	1.29	1.13	0.97	1.33
Autumn	1.16	0.99	1.36	1.17	0.99	1.37
Winter (rainy season)	**1.25**	1.06	1.47	**1.28**	1.08	1.51
**Size at birth**						
Average or larger than average (ref)	1.00					
Smaller than average	0.93	0.81	1.05			
**Birth Order**						
1st-2nd (ref)	1.00					
3rd	**0.67**	0.55	0.81			
4th+	**0.76**	0.67	0.85			
**Preceding birth interval**						
Not applicable (only one birth)	1.97	1.71	2.25	1.93	1.65	2.27
<2 years	**2.10**	1.79	2.37	**2.19**	1.89	2.52
2+ years (ref)	1.00			1.00		
**Age of mother at birth**						
<18 years	**2.08**	1.74	2.50	**1.41**	1.15	1.72
18-34 years (ref)	1.00			1.00		
35+ years	1.11	0.94	1.29	1.29	0.97	1.52
Maternal health service:						
**Tetanus toxoid injection**						
None or 1 TTI (ref)	1.00					
2+ TTI	**0.43**	0.35	0.52	**0.44**	0.36	0.54
**Delivery place/assistance**						
Home delivery/not assisted by TBA/HW	1.00					
Home delivery - assisted by TBA/HW	0.96	0.84	1.10			
Government hospital	0.95	0.74	1.21			
Health center	1.11	0.82	1.52			
Other health facility	0.74	0.45	1.24			
**Mode of delivery**						
Non-caesarian section	1.00					
Caesarian section	1.26	0.86	1.85			

*Adjusted and unadjusted HRs in bold represent statistical significance at p-value<0.05 and the corresponding 95% CIs do not include the null value 1.

Contextual factors included in the analysis were urban–rural residence and region. While the gross effect of urban–rural residence on neonatal death waned in the multivariate analysis, the effect of region was retained. Compared with neonates in Addis Ababa, those in Amhara (adjusted HR: 1.88; 95% CI: 1.26 − 2.83), Benishangul Gumuz (adjusted HR: 1.75; 95% CI: 1.15 − 2.67) and Tigray (adjusted HR: 1.54; 95% CI: 1.01 − 2.34) regions carried a significantly higher risk of dying. However, no similar excess net risk was noted for the other regions.

Being born to a mother with secondary or higher schooling was associated with a 32% decreased risk of neonatal death compared with mothers with no education. Primary education of the mothers had no protective effect against neonatal mortality. Both marital status and household wealth were associated with neonatal death in the univariate analysis but their individual effects were erased in the multivariate analysis after adjusting for the proximate factors.

The influence of proximate factors related to the child's inherent characteristics such as child's sex; size at birth; birth order; birth interval; maternal age; and season of birth were assessed using both univariate and multivariate analysis. With the exception of child's size at birth, the proximate factors were associated with an increased risk of neonatal death in the multivariate analysis. Males were 38% more likely than females to die during the neonatal period. The risk of neonatal death was 2.2 times higher among live-born infants born less than 2 years after the preceding child compared with those born at an interval of 2 or more years. Births to younger mothers aged less than 18 years were associated with a higher risk of death. The risk of neonatal death was 41% higher for births to younger mothers compared with births to mothers aged 18–34 years. There was no significant association with older maternal age (35+ years).

Our analysis also found an increased risk of neonatal death associated with births in winter. In a multivariate analysis that controlled for several factors, compared with those born in spring, births in winter carried a 28% excess risk of dying in the neonatal period. Other seasons did not carry a significant risk of dying.

The protective effect of TTI was apparent in the multivariate analysis and neonatal death risk decreased by 56% for births to mothers who received two or more TTIs before birth.

Because of significant interaction effects between urban–rural residence and place of delivery, we examined the effect of place of delivery on the risk of neonatal death separately for the urban and rural samples (Table 3). In the overall sample (Table 2) as well as in the urban area (Table 3), we found no significant association between place of delivery and the risk of neonatal death both in the univariate and multivariate analyses. However, and contrary to the widely held expectation, deliveries in government hospitals and health centers carried a higher risk of dying compared with home deliveries in the rural area. In a separate multivariate analysis of the rural sample, babies delivered in government hospitals and health centers had a 2.3 and 2.1 times higher risk of dying in the neonatal period, respectively, compared with home deliveries. However, this finding should be interpreted with caution because of the possibility of a selection bias that could arise as a result of the referral of women with severe pregnancy complications to these facilities. Of note, only 2% of the births from the rural area included in our analyses occurred in government hospitals or health centers.

**Table 3:** Cox proportional hazard models estimating Ethiopian neonatal mortality by place of delivery and residence

	**Rural (n = 27036)**			**Urban (n = 5006)**		
**Delivery place/assistance**	Adjusted HR*	**95% CI**		Adjusted HR*	**95% CI**	
Home delivery/not assisted by TBA/HW	1.00			1.00		
Home delivery - assisted by TBA/HW	1.05	0.90	1.23	0.84	0.54	1.33
Government hospital	**2.27**	1.50	3.44	0.83	0.50	1.36
Health center	**2.07**	1.35	3.17	0.72	0.41	1.27
Other health facility	1.01	0.50	2.04	0.67	0.29	1.57

Multivariate analysis adjusted for year, region, education, sex of the child, season of birth, birth interval, age of mother, and receipt of two TTIs.

*Adjusted and unadjusted HRs in bold represent statistical significance at p-value<0.05 and the corresponding 95% CIs do not include the null value 1.

## Discussion

Our analyses show that the Ethiopian NMR declined an average of 1.9% per annum in the last 15 years, although the decline has been sluggish compared with the trend for the overall under-5 mortality. The decline has been even slower for ENM, at 0.9% per annum where the majority (74%) of the neonatal deaths took place. During the same period, under-5 mortality in Ethiopia halved from 166 to 88 per 1000 live births [9]. Our analysis showed that a much faster reduction in neonatal mortality is crucial to increase child survival and achieve the MDG 4 target for the under-5 mortality rate for Ethiopia. Worldwide data also show that although most parts of the world have experienced a reduction in neonatal mortality, the trend has been slow at 1.7 per annum. From 1990 to 2009, neonatal mortality decreased from 33.2 to 23.9 per 1000 live births. In most African countries, the rate of decline has been slow; from 1990 to 2009, the average rate declined from 43.6 per 1000 in 1990 to 36.0 per 1000 in 2009 [10].

We examined the factors associated with the risk of neonatal death and multivariate analyses showed that excess neonatal mortality risk was associated with a number of proximate factors including being male, being born to younger mothers (< 18 years of age), and being born within 2 years of the last birth. Neonates born in winter had an increased risk of dying compared with those born in other seasons. We also showed a protective role if mothers received two TTIs before birth. Contrary to the usual expectation, we found no evidence of lower neonatal mortality associated with institutional delivery in the overall sample. Because of interaction between urban–rural residence and place of delivery, we used a separate multivariate model for the rural and urban areas, which showed a higher mortality risk associated with delivery in government hospitals and health centers in the rural areas. We found no association between institutional delivery and neonatal mortality in the urban areas. Women's education remained the single most important socioeconomic factor associated with neonatal mortality in the multivariate analyses. Regional variations in the risk of neonatal mortality were also apparent, even after adjusting for several maternal and child-related factors. The gross effect of urban–rural residence was erased in the multivariate analyses.

We confirmed that male children have a 38% higher risk than females of dying during the neonatal period. Previous studies confirm this, but with varying excess odds [19,23-27]. Contributing factors include immunodeficiency, higher prevalence of respiratory and other infectious diseases, and congenital malformations of the urogenital system [28,29].

Studies consistently show a relationship between short birth interval and heightened neonatal mortality risk [19,27,28]. We found that children born within a preceding interval less than 2 years were 2.2 times more likely to die during the neonatal period than those born at an interval of 2 or more years. A preceding birth interval of less than 2 years was reported to carry a 2.8 times higher risk of dying among neonates in Indonesia [19,27,28]. Analyses of DHS data from the developing world, controlling for potential confounders, indicated that neonatal mortality decreases by approximately 40% for preceding birth intervals of 3 years or more, compared with intervals of less than 2 years [29]. An approximately 20-year longitudinal study of 145 000 pregnancy outcomes from an experimental setting in Matlab, Bangladesh found that compared with intervals of 3 or more years, preceding inter-birth intervals of less than 24 months were associated with significantly higher risks of early neonatal mortality [30]. The shorter birth interval effect could be related to maternal depletion syndrome and resource competition between siblings, in addition to less care and attention experienced by high-ranked infants [31,32]. While the negative effect of shorter birth spacing on neonatal and infant mortality as well as maternal health is well established, over a fifth of Ethiopian women are currently giving birth within 2 years of a preceding birth and 16% have an unmet need for child spacing [9]. Data from the three DHS show a lack of improvement in birth interval. In 2000, 19.7% of women had a preceding birth interval of less than 2 years and the 2005 and 2011 levels were 21.3% and 20.4%, respectively. This is despite the recent surge in family planning from 8% in 2000 to 29% in 2011, and raises concerns about the effectiveness of the family planning program in addressing the unmet need for spacing.

Controlling for several confounders, maternal age of less than 18 years carried a 41% higher risk of neonatal mortality compared with maternal age of 18–34 years. Previous studies reported similar findings [33,34]. After adjustment for confounders, there was a 53% excess risk of neonatal mortality among infants born to mothers in the youngest vs. oldest age category in Nepal [34]. The higher risk of neonatal mortality among adolescent women in our setting can be due to a number of unmeasured factors. Previous studies have reported that preterm birth or small-for-gestational-age are possible mediating factors [34], neither of which was measured in our study. It is also possible that perceived young mothers’ inexperience regarding childcare works against the health and survival of their children [35].

Few studies from industrialized countries have examined the association between season and neonatal mortality. Studies from Italy [36] and the United Kingdom [37-39] show an increased risk of neonatal mortality in winter as in our study, after we controlled for proximate factors. We cannot compare our finding with the literature because of a lack of data from other similar resource-poor settings. Nevertheless, we hypothesize that the heightened neonatal mortality risk in winter in our study could be due primarily to hypothermia. A study in Northern India found that newborns born at home had an incidence of hypothermia of 19.1% in the winter months compared with 3.1% for those born in the summer [39]. Hypothermia is increasingly recognized as a major cause of neonatal morbidity and mortality in resource-poor settings [40]. The World Health Organization (WHO) adopted thermal control among the essential components of newborn care [41] and provided guidelines for “warm chain” steps to minimize the risk of hypothermia [42]. In Ethiopia, intervention efforts that promote essential newborn practices including thermal control and dispelling some cultural practices, such as early bathing and not drying or unwrapping the baby before the placenta delivers, remain to be improved in the rural population where over 90% of the deliveries took place at home.

The protective role of maternal TTI was a strong finding in our analysis. The neonatal mortality risk decreased by 56% in neonates whose mothers received two tetanus injections prior to delivery compared with neonates whose mothers did not receive two tetanus injections, similar to reports from Kenya, Bangladesh and Indonesia [35,43,44]. Neonatal tetanus remains an important and preventable cause of neonatal mortality globally. Large reductions in neonatal tetanus deaths have been reported following increases in tetanus toxoid immunization. According to the Lancet Neonatal Survival Series, in settings where tetanus remains an important cause of neonatal deaths, delivering tetanus toxoid immunization to the mother can result in a reduction in neonatal mortality of 33 − 58% [11]. In Ethiopia, tetanus has been identified as a major cause of neonatal mortality [7,45], and immunization of pregnant women during antenatal visits and outreach services has been part of the primary healthcare package. Nevertheless, according to the 2011 DHS, only 33.8% of pregnant women received at least two TTIs, although this has increased from a low of 17.2% in the 2000 DHS [9].

In the overall as well as urban samples, we found no significant association between place of delivery and neonatal mortality. Contrary to the widely held expectation, however, increased neonatal mortality risk was associated with deliveries in public hospitals and health centers compared with home deliveries in the rural area. Similar findings have been reported in other countries [18,46]. Only 2% of the rural neonates included in this study were delivered in public hospitals/health centers, while the vast majority (over 97%) was delivered at home. Deliveries in private and other facilities represented < 1% of all deliveries. One explanation for this increase is a selection bias that could arise due to the referral of pregnant women with severe pregnancy complications to these facilities [28]. Even without this bias, the excess mortality in public facilities also raises concerns about the quality and preparedness of the emergency obstetric and newborn care services. Socioeconomically, maternal education (secondary or higher level of schooling) had a protective effect against neonatal mortality in our multivariate analysis. However, the gross influence of household wealth and marital status disappeared in the multivariate analysis. The positive effect of maternal education on the survival of children, including neonates, is well supported worldwide [6,19,47-50]. A common supporting argument is that maternal education increases mothers’ knowledge about child health and healthcare services [48], and thereby improves mothers’ healthcare-seeking behaviors for their children and themselves [51]. In our study, the influence of maternal education on neonatal mortality did not change even after controlling for proximate factors, suggesting the presence of unmeasured factors. Possible mediating factors not measured in our study include maternal nutrition; environmental sanitation/hygiene; antenatal service use; the adoption of essential newborn care practices; the use of postnatal care services and seeking timely health services for sick babies.

The gross effect of urban–rural residence on neonatal mortality was erased after adjustment for several proximate factors. However, some regions of the country had a greater neonatal mortality risk than others in the multivariate analysis. Compared with neonates in Addis Ababa, neonates in Amhara, Benishangul Gumuz and Tigray regions carried a significant net increased risk of dying. We cannot fully explain this finding but unmeasured mediating factors such as nutrition, environmental factors, and prevailing cultural/child care practices may play a role. Further investigation is needed to identify specific factors that are relevant to these regions. Identifying such factors will help programmers to design and implement region-specific interventions to better address neonatal mortality.

This study has notable strengths. The data for this study are drawn from the 2000, 2005 and 2011 Ethiopia Demographic and Health Surveys, based on nationally representative probability samples. By combining the data from the three surveys, we achieved a large sample size that allowed us to estimate the effects of several factors on neonatal mortality with acceptable precision. The three DHS also permitted investigation of neonatal mortality trends over the past 15 years because the surveys employed similar sampling methodologies and questionnaires, used comparable data collection tools, and they were organized and implemented by the same institutions. Another strength of the study is that the DHS 5-year birth history data provide good to excellent information to estimate child mortality [21,52], and this has been shown to reduce recall errors in the reporting of birth and death dates by the interviewed mothers [53,54]. Our assessment of data quality in this paper also concluded that there was a lack of serious omission of births and deaths. The relative sampling errors for the neonatal mortality estimates by single year from 1995–2011 suggested that the estimates for each year are within the acceptable standard error.

The study also has several limitations. First, we cannot completely rule out the possibility of underreporting of the deaths of neonates by mothers or caretakers despite our data quality assessment. Mothers may avoid reporting neonates who died in the first few hours after birth for social or cultural reasons. Second, only surviving mothers were interviewed in the DHS and there could be some association between maternal death and neonatal mortality that could result in underestimation of neonatal deaths. Third, information on certain important predictors of neonatal mortality such as antenatal care use were collected only for the most recent delivery, which restricted our evaluation of the role of such variables. Postnatal care use was not included in our analysis because the majority of neonatal deaths occur in the first few hours after birth. Fourth, because of a lack of data, we did not evaluate the roles of other determinants such as essential newborn care practices and environmental factors.

## Conclusions

Our analyses show that the current declining trend in neonatal mortality in Ethiopia is inadequate to achieve the MDG 4 target of reducing the under-5 mortality rate by two-thirds by the year 2015. A much faster decline in neonatal mortality is needed. The study also underscores that strategies to address neonatal survival require a multifaceted approach that encompasses health-related measures as well as several other measures.

Addressing short birth interval and preventing early pregnancy and child birth through effective family planning programs need to be considered among neonatal survival interventions. Improving the coverage of maternal TTI through antenatal care and outreach services should be given high priority, as this benefits both the child and the mother. There is also a need for community-based prevention, recognition, and management of hypothermia by educating mothers and community workers, and this effort should give proper emphasis to births in winter. Even without a possible selection bias, the higher neonatal mortality risk in rural areas that was associated with delivery in public facilities necessitates appraisal of the quality and preparedness of the emergency obstetric and newborn care services in such facilities.

Women's education is a cornerstone for improved health outcomes, and we have shown that secondary education for women is essential to improve neonatal survival. While improving women's education is a long-term strategy, short-term community-based interventions to educate and counsel mothers and caretakers to improve pregnancy outcomes and neonatal survival is of paramount importance. Any strategy targeted at addressing neonatal survival in Ethiopia must consider regional variations by incorporating specific risk factors that are relevant to the different regions.

## Abbreviations

CI: Confidence interval; DHS: Demographic and Health Survey; ENMR: Early neonatal mortality rate; HR: Hazard ratio; LNMR: Late neonatal mortality rate; MDG: Millennium Development Goal; NGO: Non-Governmental Organization; NMR: Neonatal mortality rate; RSE: Relative standard error; TBA: Traditional birth attendant; TTI: Tetanus toxoid injection; US: United States; WHO: World Health Organization.

## Competing interests

The authors declare that they have no competing interests.

## Authors’ contributions

YM conceived and designed the study, analyzed the data and wrote the manuscript. BT contributed to the acquisition of data, analysis and drafting of the manuscript. DS, TD and AB were involved in the interpretation of the data and contributed to manuscript preparation. YM, BT, DS, TD and AB approved the final manuscript.

## Authors’ information

YM holds a PhD in epidemiology and MSc in demography. YM has 20 years’ experience in public health research with particular emphasis on reproductive health, maternal and child health. YM has authored and co-authored over 30 scientific articles in reputable journals and is currently serving as the Managing Director and Senior Researcher for Mela Research PLC in Ethiopia.

BT holds an MSc in demography with more than 10 years’ experience in management and analysis of longitudinal and cross-sectional data. BT has authored and co-authored a number of scientific articles in reputable journals and is currently serving as a senior data management specialist for the Saving Newborn Lives Program within the Health and Nutrition Unit of Save the Children, USA in Ethiopia.

DS holds PhDs in international development and social demography and has over 25 years’ experience in research, teaching and program evaluation in Ethiopia and Canada. DS is affiliated with Mela Research PLC as a Senior Research Associate.

TD holds an MD and a Master's Degree in public health (MPH) and has more than 20 years’ experience in community-based child health interventions. She has also led a number of child survival programs in the country and is currently working for UNICEF as a Health Expert in the Child Survival Program.

AB holds an MD, a Master's Degree in public health (MPH) and an MA in medical anthropology. AB has over 20 years’ experience in managing health programs in governmental, non-governmental and multilateral organizations. Currently, AB is serving as Health and Nutrition head of the Save Newborn Lives program in Ethiopia under Save the Children USA.

## Pre-publication history

The pre-publication history for this paper can be accessed here:

http://www.biomedcentral.com/1471-2458/13/483/prepub
